# D2 receptors and cognitive flexibility in marmosets: tri-phasic dose–response effects of intra-striatal quinpirole on serial reversal performance

**DOI:** 10.1038/s41386-018-0272-9

**Published:** 2018-11-15

**Authors:** Nicole K. Horst, Bianca Jupp, Angela C. Roberts, Trevor W. Robbins

**Affiliations:** 1 Department of Psychology, University of Cambridge, Downing Street, Cambridge, CB2 3EB UK; 2 Behavioural and Clinical Neuroscience Institute, University of Cambridge, Downing Street, Cambridge, CB2 3EB UK; 3 Department of Physiology, Development and Neuroscience, University of Cambridge, Downing Street, Cambridge, CB2 3DY UK

**Keywords:** Cognitive control, Reward

## Abstract

Behavioral flexibility, which allows organisms to adapt their actions in response to environmental changes, is impaired in a number of neuropsychiatric conditions, including obsessive-compulsive disorder and addiction. Studies in human subjects and monkeys have reported correlations between individual differences in dopamine D2-type receptor (D2R) levels in the caudate nucleus and performance in a discrimination reversal task, in which established contingent relationships between abstract stimuli and rewards (or punishments) are reversed. Global genetic deletion of the D2R in mice disrupts reversal performance, indicating a likely causal role for this receptor in supporting flexible behaviors. To directly examine the specific role of caudate D2-type receptors in reversal performance, the D2/3/4R agonist quinpirole was infused via chronic indwelling cannulae into the medial caudate of male and female marmoset monkeys performing a touchscreen-based serial discrimination reversal task. Given prior evidence for dose-dependent effects of quinpirole and other dopaminergic drugs, a full dose-response curve was established. Individually, marmosets displayed marked differences in behavioral sensitivity to specific doses of intra-caudate quinpirole. Collectively, they exhibited a behaviorally specific bi-phasic deficit in reversal learning, being consistently impaired at both relatively low and high doses of quinpirole. However, intermediate doses of intra-caudate quinpirole produced significant improvement in reversal performance. These data support previous human and monkey neuroimaging studies by providing causal evidence of a U-shaped function describing how dopamine modulates cognitive flexibility in the primate striatum.

## Introduction

The ability to adapt one’s actions following changes in the relationship between a stimulus in the environment and its associated outcome is a central component of cognitive flexibility. Dysfunction in this domain is evident in conditions like obsessive-compulsive disorder (OCD) and addiction [1], wherein habit-like, compulsive behavior supersedes normal goal-directed behavior [2]. Such behavior can be modelled experimentally using a reversal task, wherein a previously learned stimulus-outcome association is reversed, and subjects must desist responding toward the formerly rewarded stimulus to redirect their responses toward a stimulus with no recent association with reward.

The caudate nucleus of the striatum is a critical locus supporting the successful reversal of stimulus-outcome contingencies. Event-related imaging has provided correlational evidence of enhanced activity within this subcortical region during reversal paradigms in healthy human subjects [3, 4]. Ablative lesions of the caudate in rhesus macaques [5] and excitotoxic lesions in marmosets [6] produce profound perseverative reversal impairments. Lesions or reversible inactivations of the homologous striatal region—the dorsomedial striatum—also disrupt reversal learning in rats [7–10]. Crucially, anatomical and functional differences have also been identified in this region in OCD patients [11, 12] and stimulant abusers [13].

**Fig. 1:**
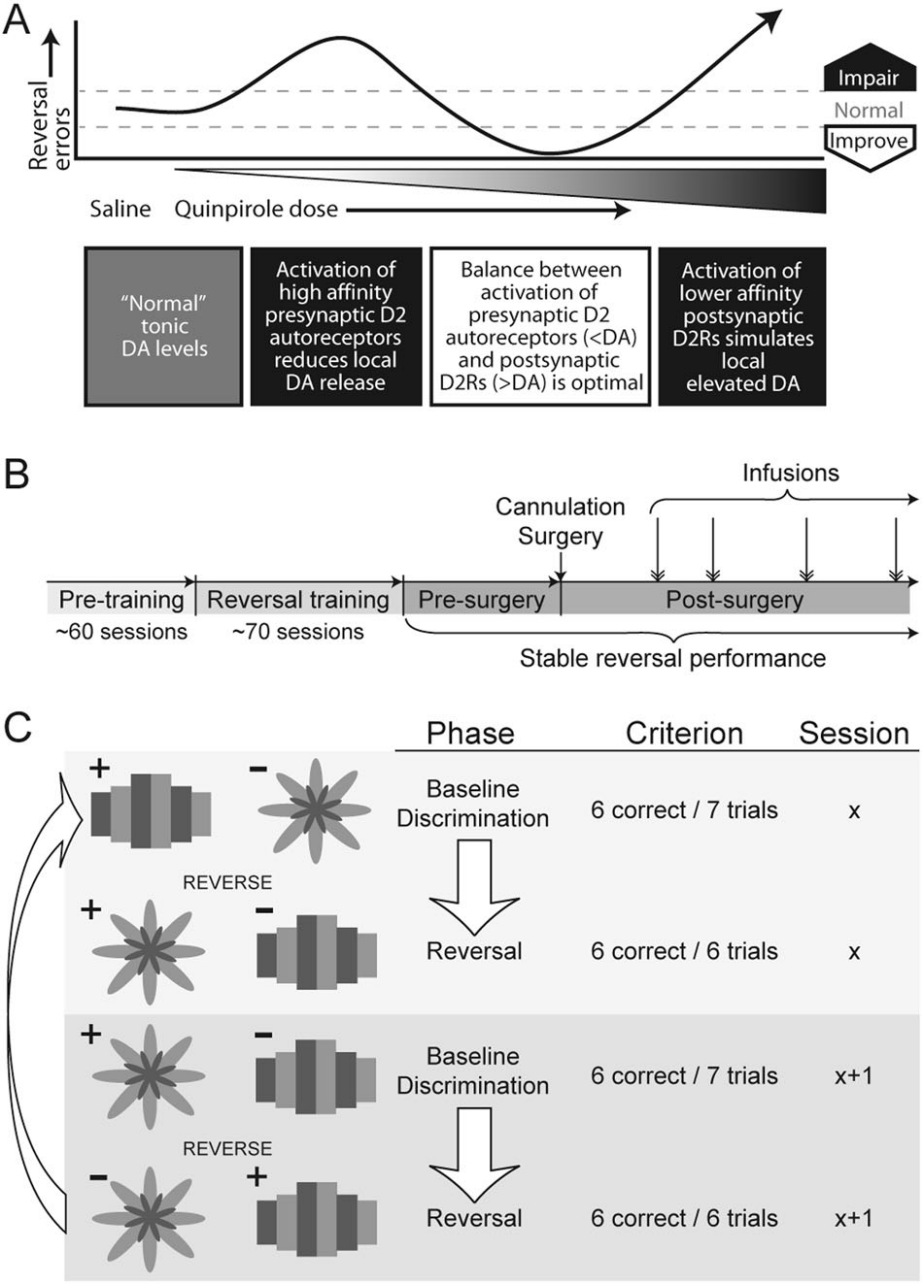
Behavioral training and testing. **a** Hypothetical dose–response curve for the potential tri-phasic effects of intra-caudate quinpirole on reversal performance. At the high-dose end of the curve, elevation of dopamine is likely to cause non-specific behavioral disruption as well as increased reversal errors. **b** Experimental timeline. See “Behavioral Training” in “Supplementary Materials and Methods” for detailed descriptions of the training phases. **c** Within-session discrimination reversals. At the start of each session (“*x*”), marmosets learned by trial-and-error that responding to one of two presented stimuli was associated with reward delivery, while response on the other stimulus produced negative auditory feedback. The relative position of visual stimuli varied across trials in a pseudorandom fashion, with each individual stimulus presented equally to the left or right of the reward licker across the session. Once criterion in this “baseline discrimination” phase was reached (6/7 correct trials), these stimulus-outcome associations were reversed. Animals were considered to have successfully reversed these contingencies upon achieving the reversal criterion (6/6 correct trials). In the next session (“*x* + 1”), the stimulus that had been rewarded on the reversal phase of the preceding session (“*x*”) remained rewarded in the baseline discrimination phase. Provided reversal criterion was reached in session “*x* + 1”, session “*x* + 2” would revert to the same initial stimulus-outcome contingencies as session “*x*”, and so on. Pharmacological manipulations were performed when marmosets were reliably performing daily within-session reversals

Caudate activity is strongly modulated by dopamine, and dopaminergic manipulations have been shown to affect reversal performance. Thus, systemic amphetamine administration, which elevates synaptic dopamine, induced perseverative performance after object-outcome reversal in marmosets [14], whereas selective depletion of caudate dopamine in marmosets [15] and rats [16] also impaired reversal. These findings are consistent with an inverted U-shaped function relating striatal dopamine to reversal learning. Klanker et al. [17] summarized data from multiple systemic psychostimulant studies that together describe such a curve, with low and high doses producing impairments and intermediate doses either improving or having no effect on reversal performance. Hypothetically, this pattern arises from a combination of inhibitory presynaptic D2 autoreceptors on dopaminergic striatal terminals which inhibit dopamine release (or on the cell bodies of midbrain dopamine neurons which inhibit dopamine cell firing), in combination with post-synaptic D2 receptors (D2Rs) of lower affinity for D2R agents that mimic elevated dopamine levels at D2-type receptors [18] (Fig. 1a).

Striatal D2Rs do appear to play an important role in reversal learning. Marked individual differences in performance were associated with D2R levels in the caudate nucleus, revealed by a genetic polymorphism associated with reduced striatal D2R expression in human subjects that correlated with impaired reversal learning [19]. The degree of displacement of the radiolabeled D2-type antagonist raclopride in the caudate by systemic administration of the psychostimulant methylphenidate predicted reversal performance and accounted for variance between healthy human subjects [20]. In addition, vervet monkeys with greater D2-type receptor availability were better at reversing stimulus-outcome contingencies [21], and viral-mediated shRNA knockdown of D2R, but not D1R, expression in the caudate disrupted reversal performance of marmosets [22]. Further evidence arising from systemic administration of the D2-type antagonist raclopride indicates a role for the D2-type receptors in reversal learning in v ervet monkeys [23], although such effects could implicate prefrontal cortex [24], as well as the striatum.

Whilst these data provide compelling evidence for the contribution of D2-type receptors to reversal learning, further investigation is required to determine the neural basis of these effects. Thus, the D2/3/4R agonist quinpirole was administered via chronically implanted cannulae into the medial caudate of marmosets performing a serial reversal paradigm that allowed for multiple within-subject infusions. Because of the existence of inhibitory presynaptic D2Rs we hypothesized tri-phasic effects of intra-caudate quinpirole on behavioral performance (Fig. 1a) and hence explored full dose–response curves in individual subjects, accounting for potential inter-individual variability (as in [25]).

## Materials and methods

### Subjects and housing

Six common marmosets (*Callithrix jacchus;* two females, four males) participated in this study and had restricted access to water to motivate performance. All procedures were carried out in accordance with the UK Animals (Scientific Procedures) Act 1986 as amended in 2012, under project licences 80/2225 and 70/7618. In addition, the University of Cambridge Animal Welfare and Ethical Review Body provided ethical approval of the project licence and its amendments, as well as individual studies and procedures via delegation of authorization to the Named Animal Care and Welfare Officer for individual study plans. For further details on husbandry and welfare, see Supplementary Materials and Methods.

### Behavioral training

#### Serial discrimination reversal

Figure 1b shows the experimental timeline for behavioral training, testing, cannulae implantation, and infusions. For complete details of behavioral training see Supplementary Materials and Methods.

In order to compare the selectivity of any reversal effects of multiple doses of a D2-type agonist infused into the caudate nucleus, animals were trained to perform a “baseline discrimination” and “reversal” in each session. Inclusion of a baseline discrimination phase allowed us to determine the effects of experimental manipulations on the reversal per se, independent of any potential impact on visual discrimination performance. Having reached consistent performance on this schedule animals were cannulated, re-tested for stable performance, and then infusions began. The same pair of multi-coloured patterned stimuli was used throughout. In the “baseline discrimination” phase, marmosets were presented with these two abstract, multi-colored stimuli on a touchscreen and learned, by trial and error, to select the correct one to gain access to banana-flavored milk reward (Fig. 1c). Erroneous responses on the alternative stimulus were penalized with a 0.25 s, 100 dB auditory negative reinforcer followed by a 5 s timeout, during which the house light was extinguished. The stimulus-reward contingency reversed after attaining a criterion of six correct responses in seven trials. In the subsequent “reversal” phase, the previously correct stimulus became incorrect and the previously incorrect stimulus became correct. Animals met the criterion for successful reversal by making six consecutive correct responses. In the next session, the stimulus that had been rewarded on the reversal phase of the preceding session remained rewarded in the baseline discrimination phase of the next session. On the rare occasion when animals failed to reach reversal criterion within a session, usually as a consequence of a drug infusion, the same contingencies were in operation the following session and for however many sessions were required until they successfully re-attained criterion. A side-bias correction procedure was active throughout training and testing but was rarely triggered once animals were fully trained. A session terminated after whichever of the following came first: reversal criterion was reached, failure to respond for over 2 min, or after 30 min of testing.

### Cranial cannulation surgery

**Fig. 2:**
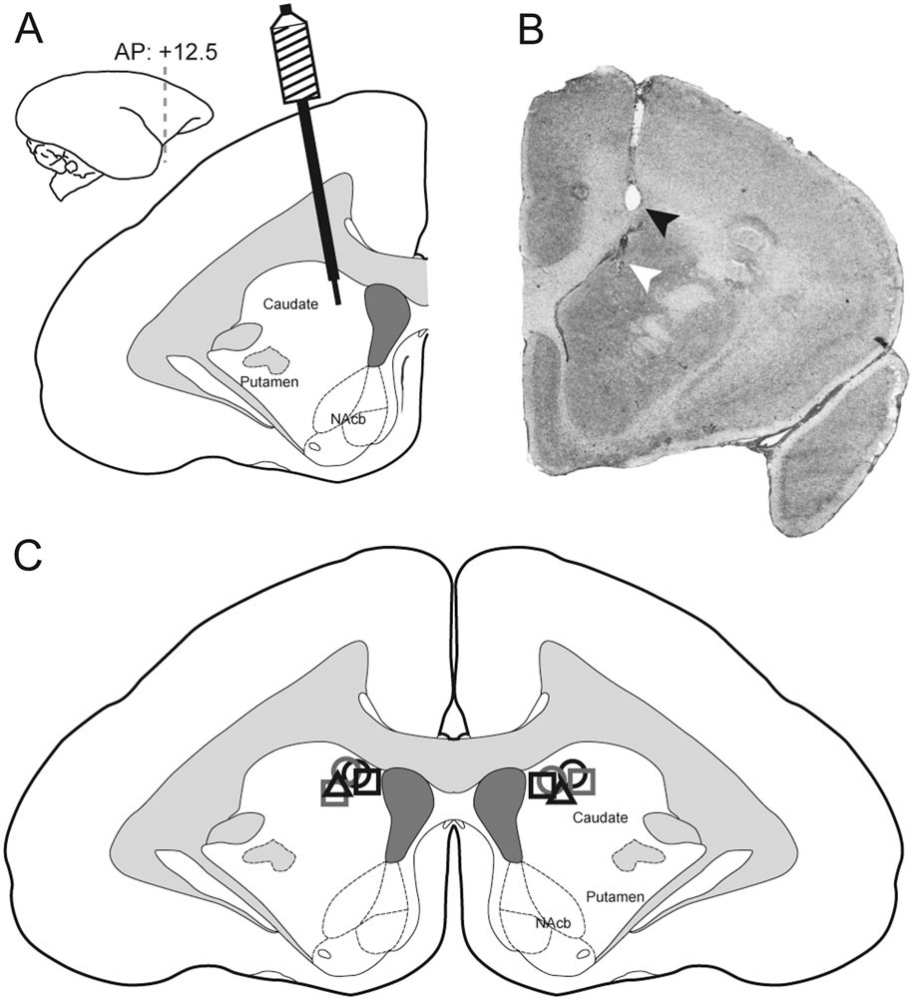
Anatomical localization of intra-striatal cannulae. **a** Marmosets were implanted bilaterally with guide cannulae targeting the medial caudate. **b** Cresyl-stained coronal section from a marmoset implanted with intra-striatal cannulae. The black arrow indicates the end of the guide cannula. The white arrow denotes the tip of the injector cannula. There was little sign of any non-specific tissue damage around the cannula tips. **c** Composite image showing locations of cannula tips. Schematics adapted from Paxinos et al. [56]. Cannulae were all located in close proximity within the medial caudate nucleus. Striatal regions are labelled for reference; NAcb = nucleus accumbens

For a detailed description of surgical procedures, see Supplementary Materials and Methods. Briefly, guide cannulae (C316G; Plastics One, Inc., Roanoke, VA, USA) were lowered at a 10° lateral angle (away from the midline), toward the target locations in medial caudate (Fig. 2a; AP +12.5, LM ±2.2, V +12.0 from the interaural line).

### Autoradiography

Following completion of experiments, marmosets were euthanized and their brains removed and flash frozen. Triplicate 20-μm coronal sections incorporating the infusion target area were collected and mounted on slides to undergo autoradiographic D2-type and D1-type receptor binding [26] (Figure S2; Supplementary Materials and Methods).

### Histological assessment of cannulation placements

For verification of cannulae placement, appropriate sections were stained with Cresyl Fast Violet to visualize cell bodies (Fig. 2 and Supplementary Materials and Methods).

### Intracranial infusion procedure

After recovery from surgery, behavioral sessions resumed and animals were habituated to infusion procedures (Supplementary Materials and Methods). Marmosets then received infusions of either quinpirole (quinpirole hydrochloride, Sigma-Aldrich, Co., St. Louis, MO, USA; 0.5 µl of 0.006–20.0 µg/µl in saline, adjusted to pH ~7.0 and filtered for sterility) or sterile saline vehicle (0.5 µl) under aseptic conditions. Infusions lasted 2 min and internal cannulae were left in place for 1 min after the infusion so as not to draw infused solution back into the guide. Injections were confirmed by monitoring movement of a small air bubble separating the drug from saline solution in the syringe/tubing assembly (Supplementary Materials and Methods). Animals were returned to their home cage and were tested ~5 min after the end of the infusion procedure.

### Experimental design and statistical analysis

#### Quinpirole administration regimen

Initial doses of quinpirole (0.3, 1.0, and 3.0 µg per side) were selected based upon their ability to enhance locomotor activity when infused bilaterally into the medial caudate (Supplementary Materials and Methods). Animals displayed marked individual variation in behavioral sensitivity to these initial doses, resulting in the dose range being extended to include both lower and higher doses in order to cover the entire dose–response curve for each individual. Doses were repeated in some cases for confirmation, or, in the case of saline, when a substantial period of time had elapsed between control infusions. Marmosets received 1 or 2 infusions per week depending upon the stability of their performance; infusions were given only after two sessions of stable performance within each individual’s normal range. Infusions were administered at least 48 h apart from one another, separated by a standard, non-infusion session. The order of infusions for each animal is provided in Table 1. One animal did not complete a full dose–response curve; Subject 6 sustained irreparable damage to the implant and did not receive the highest possible dose.

**Table 1:** Order of quinpirole and saline infusions by subject

Subject	Infusion number and dosage (in μg per side)									
	1	2	3	4	5	6	7	8	9	10
1	1.0	3.0	0.3	0.0	0.03	3.0	10.0	–	–	–
2	0.0	3.0	1.0	0.3	10.0	0.003	0.03	0.0	–	–
3	1.0	0.0	0.3	3.0	0.0	–	–	–	–	–
4	6.0	1.0	10.0	0.0	1.0	3.0	0.3	0.03	0.003	0.3
5	0.0	0.3	3.0	1.0	0.1	0.015	10.0	0.003	0.0	–
6	0.0	3.0	1.0	0.3	0.003	0.0	–	–	–	–

#### Statistical approach and data analysis

**Fig. 3:**
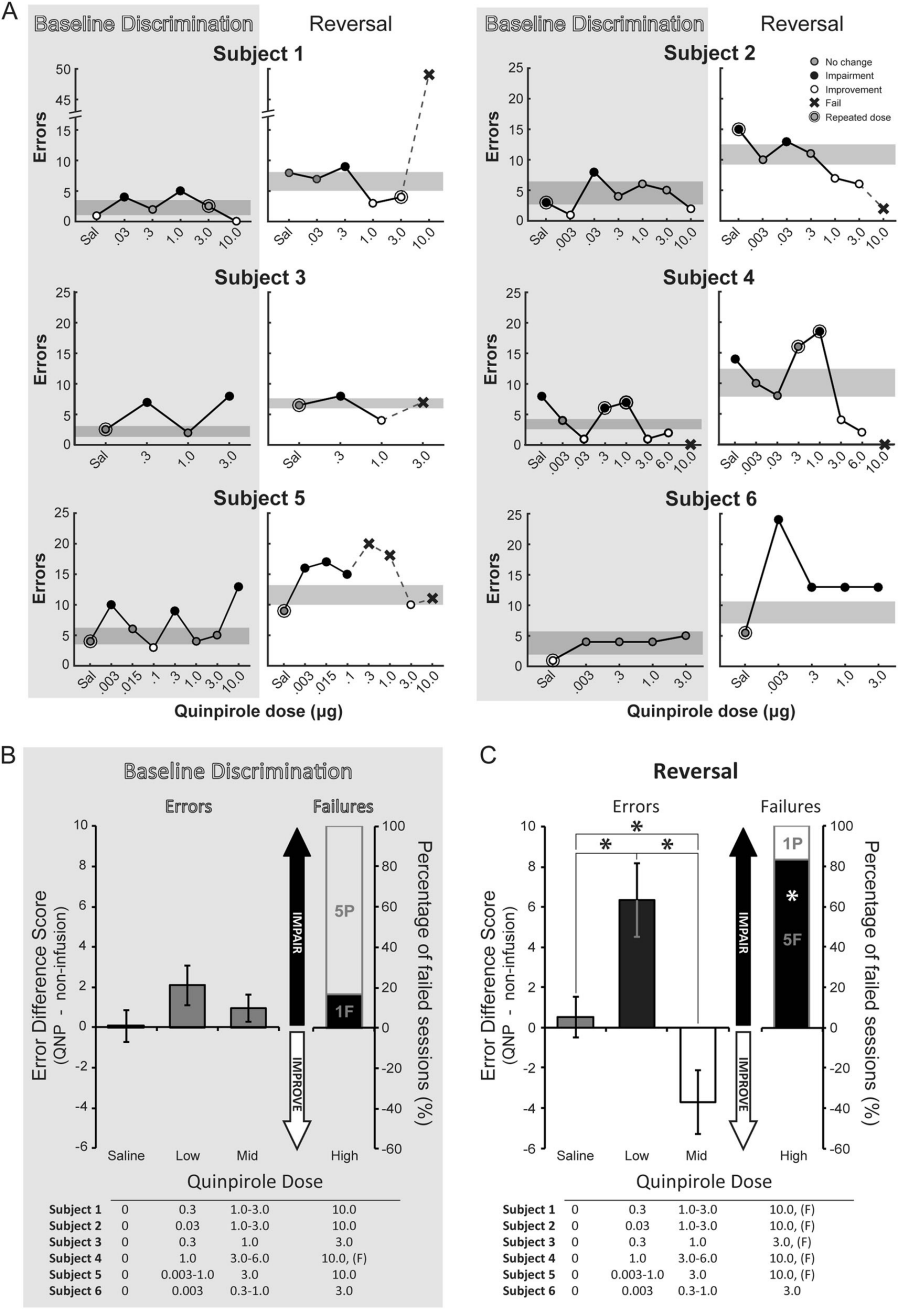
Individual and group analyses of error data suggest tri-phasic effects of intra-caudate quinpirole. **a** Individual dose–response curves of numbers of errors during baseline discrimination (shaded area) and reversal (unshaded area) phases for each infusion (circles). The 95% confidence interval (horizontal grey band) is calculated for each task phase from the errors committed in the preceding, non-infusion sessions (two control sessions for each infusion). Any point lying outside of this confidence interval band is significantly different from stable performance. Circles are color-coded to indicate no difference from control sessions (grey), impairment (black), or improvement (white). A black “X” denotes failure to reach criterion within the task phase (either due to premature cessation of responding/non-performance or reaching the session duration limit). Cases in which a particular dose was given on multiple occasions, and its effects averaged, are illustrated by a larger circle around the primary filled circle. Subjects 1–5 were improved during reversal by intra-caudate quinpirole at a mid-range dose. Subject 6 never showed a reversal improvement at any dose, but did not complete a full dose–response curve due to irreparable implant damage. **b** and **c** Group summary and analysis of tri-phasic intra-caudate quinpirole effects showing low and high dose impairments and intermediate dose improvements in baseline discrimination (**b**) and reversal (**c**) phases. Significant effects of quinpirole in individual animals were categorized for group analysis as follows: low-dose reversal impairments, mid-dose reversal improvements, and more generalized high-dose impairments/failures. Actual doses included in each dose category are shown in the tables below the bar plots. Low- and mid-dose effects were compared against the saline control infusion in a two-factor mixed model ANOVA. The dependent variable was a difference score between errors on the infusion day minus the mean errors of two preceding non-infusion sessions. There was a significant dose-dependent effect of intra-caudate quinpirole on reversal, but not baseline discrimination performance, with low-dose quinpirole inducing more errors and mid-dose reducing errors committed. A Fisher’s Exact Test was used to compare of the proportions of animals that failed to reach criterion at the high dose vs. saline in both task phases. (Black * = *p* < 0.05 by general linear hypothesis test following two-factor mixed model ANOVA. White * = *p* < 0.05 by Fisher’s Exact Test of the proportion of failures vs. successes during the baseline discrimination and reversal following high-dose quinpirole vs. saline. Note that all animals passed reversal criterion after saline infusions.)

The primary measure of behavioral performance used for analysis was the number of errors made before reaching criterion. Trial counts, response latencies, and failure to complete either the baseline discrimination or reversal phases were also recorded. Since the behavioral performance of individual marmosets was differentially sensitive to specific doses of quinpirole, the first stage of analysis used dose–response curves for each subject to visualize the numbers of errors committed for both baseline discrimination and reversal phases (Fig. 3a). To assess significant deviations from typical performance produced by quinpirole in individual marmosets, 95% confidence bands were constructed from the set of non-infusion sessions (the two preceding non-infusion sessions used to confirm stability for each dose of quinpirole received). The confidence intervals were calculated as the standard error multiplied by the critical two-tailed value of *t* for *α* = 0.05 for a given degrees of freedom (*n* − 1) [27]. Significant performance improvements and impairments for each individual animal are represented by circles that lie, respectively, below or above the confidence band.

In order to confirm trends in the data that appeared consistent across subjects, doses were categorized on a per-subject basis as “low”, “middle”, and “high” to facilitate group analysis. Details of this data stratification are discussed further in Results. The group analysis used an error difference score [number of errors in the infusion session − mean errors committed in the two preceding non-infusion sessions]. The effect of dose on the error difference score in the baseline discrimination phase vs. reversal was assessed using two-factor linear mixed model analysis of variance (ANOVA) with Type III sums of squares using the statistical computing language R (R x64 v3.4.1) running custom-programmed scripts via RStudio (v1.0.153). Details of the specific R packages and functions employed are included in Supplementary Materials and Methods. Task “phase” (baseline discrimination vs. reversal) and “dose” category (quinpirole doses vs. saline) were included in the model as fixed factors with “subject” contributing as a random factor.

Interactions were further investigated using one-way mixed model ANOVAs and general linear hypothesis tests with the Holm adjustment for multiple comparisons. Although analysis of error count is typically the most sensitive means of detecting performance changes in reversal paradigms like this, a complementary analysis of the trial difference score was also carried out, as differences in the degree of change in these two variables may provide greater insight into the nature of the behavioral effects.

The high dose could not be included in the mixed model ANOVA, because 4/6 animals stopped responding prematurely. Instead, the high dose impairment was assessed using Fisher’s Exact Test to determine whether the incidence of such “failures” was significantly greater following high dose quinpirole vs. saline, within both task phases.

## Results

### Behavioral results

#### Baseline performance

As can be seen from Fig. 3a, animals were performing ~3.53 ± 0.33 errors at baseline phase and 9.13 ± 0.62 errors in reversal. This performance remained stable across all infusions, as shown in Figure S3.

#### Intra-caudate quinpirole produces tri-phasic effects on reversal performance

##### Individual analysis

Inspection of the dose–response curves (Fig. 3a) revealed that marmosets were significantly impaired both at relatively low and high doses of quinpirole. At intermediate doses, performance was spared and often improved, producing tri-phasic response profiles. Subject 6 had a large, low dose deficit which was attenuated at higher doses but never received the consistently disruptive 10 μg high dose. Whilst the overall tri-phasic profile was observed in 5/6 marmosets, there was considerable individual variability in sensitivity to quinpirole. For example, the “low dose” impairment was observed across a broad range of doses (0.003–0.3 μg) and only became evident in Subject 1 at 1.0 μg, a dose which produced improvements in Subjects 2–4. In contrast, improvements in Subject 1 were only observed at the higher doses of 3.0 and 6.0 μg, which disrupted performance in Subjects 3 and 6.

The “low dose” impairments generally presented as an increase in the number of reversal errors committed, also accompanied in some animals (Subjects 2, 3, 4, and 5) by an increase in errors in the baseline phase. For the “mid dose” improvements in reversal, only Subject 4 exhibited parallel improvements in baseline performance. After “high doses” of quinpirole, animals typically failed to reach the reversal criterion, due to either premature disengagement from the task or inability to reverse in the allotted session time. Only 3/6 animals also showed disruption in the baseline phase.

In summary, all animals were significantly impaired at relatively low and high doses of quinpirole, with a majority of animals showing quinpirole-induced improvements in the mid-range of doses for reversal performance. Effects on baseline discrimination were less consistent.

##### Group analysis

To confirm the apparent tri-phasic effects of intra-caudate quinpirole at the group level, we employed a combination of a mixed model ANOVA with factors of dose and phase and a Fisher’s Exact Test to characterize the disruptive effects of the highest doses. Absolute (μg) dose values were not used as dose factor levels in this ANOVA because the consistent, ordinal effects of quinpirole did not occur at fixed doses across animals. Thus, the dose factor comprised three levels representing saline control, “low”, and “mid”, with the low dose being defined when significant impairments preceded significant improvements for Subjects 1–5 from Fig. 3a. For Subject 6, we simply defined the lowest dose as “low” and the next two ordinal doses as “mid”, since this animal did not show improvement at any dose. (Precise doses in these dosing categories for each animal are summarized in the tables below Fig. 3b, c.)

This two-way mixed model ANOVA on the error difference score revealed a significant interaction between quinpirole dose category and task phase (*F*(2,58) = 8.46, *p* = 0.0060), as well as a main effect of dose (*F*(2,58) = 12.3, *p* = 0.000035), with no effect of phase (*F* < 1). Further investigation using one-way mixed model ANOVAs on the two task phases indicated a significant effect of dose category in the reversal phase (*F*(2,27.7) = 15.1, *p* = 0.000037) but not in the baseline discrimination phase (F(1,58) = 1.09, p = 0.35). Post hoc general linear hypothesis tests on the model of reversal phase dose effects revealed that low doses of quinpirole increased (*p* = 0.0027) while mid-range doses decreased (*p* < 0.030) the error difference score as compared to saline, as well as a significant difference between low-range and mid-range doses (*p* < 1.4 × 10^−7^). These results provide evidence in support of the first two arms of the tri-phasic effects of intra-caudate quinpirole on reversal performance (Fig. 3c).

Complementary analyses of trial counts and response latencies were conducted by mixed model ANOVA to further characterize the behavioral effects of intra-caudate quinpirole. The dose-dependent pattern of trial difference scores was qualitatively similar to that seen for error difference scores, but the mid-range dose improvement was not significant. Response latencies were prolonged at the highest dose compared to all other infusions. See Supplementary Results for statistical details.

For the highest dose in each animal, separate Fisher’s Exact Tests of the proportions of disruptive failures for the baseline and reversal phases (saline vs. quinpirole) confirmed that animals were impaired selectively during reversal (*p* = 0.015 vs. baseline discrimination *p* = 1.0; Fig. 3b, c). This therefore provides evidence for the third arm of the tri-phasic quinpirole effects on reversal.

## Discussion

The main findings from this study were that the D2-like agonist quinpirole infused into the medial caudate nucleus of marmoset monkeys over a very wide dose range (0.003–10.0 μg/0.5 μl) produced tri-phasic behavioral effects, specifically on visual reversal learning performance in a computerized touchscreen paradigm: low dose impairments, mid-range dose improvements in the majority of animals, and high dose disruptions. The effects at low and intermediate doses were behaviorally selective at the group level, baseline visual discrimination performance being inconsistently impaired. There were also considerable individual differences in sensitivity to quinpirole, necessitating an experimental design that used each animal as its own control, as well as considering the group as a whole in a univariate, mixed model ANOVA. These are perhaps the first data examining behavioral effects of an intra-striatal dopamine agonist in non-human primates, especially over the full dose–response range. The findings confirm an important causal role for striatal D2-like receptors in reversal learning, as previously suggested from systemic psychopharmacological [14, 23] and PET imaging studies in monkeys [21] and humans [20], rat studies similarly employing intra-striatal infusions of the drug [28], and evidence from genetic deletion of the D2R in mice [29–31].

An important finding of the present study was the significant low dose selective impairment of reversal learning at the group level, which is hypothesized to have resulted from a reduction of dopamine release in the striatum caused by occupation of inhibitory autoreceptors on striatal nerve terminals. These results are compatible with findings that reversal learning is impaired by striatal dopamine depletion in both rodents [16] and monkeys [15]. Although somatic-dendritic autoreceptors are implicated in reductions in rodent locomotor activity caused by low doses of dopamine agonists [32], there is a paucity of evidence that such inhibitory effects could be obtained via striatal terminal dopamine autoreceptors. The precise mechanisms underlying these regulatory effects on dopamine release probably differ for the midbrain somatic-dendritic and terminal striatal autoreceptors, being mediated by the activation of the G-protein-activated inwardly rectifying potassium channels  vs. Kv1.2 channels, respectively [18, 33, 34]. The present data are consistent with effects of quinpirole administered into the dorsal striatum of rats, which produced biphasic effects on locomotor activity, similar to that observed following systemic doses, but unlike the effects of intra-nucleus accumbens quinpirole, which produced only increases in activity [35].

Low systemic doses of quinpirole have previously been shown to impair motor and cognitive performance (in a spatial delayed response working memory paradigm) in young and old rhesus monkeys, with larger deficits in younger monkeys [25]. That study also reported large individual differences in performance following quinpirole over a wide dose range (0.0001–1.0 mg/kg) that were also a feature of this study. Similar variability in dose–response sensitivity here necessitated the adoption of behavioral criteria to categorize doses as “low”, “mid-range”, or “high”, in the present study similar to that described in Arnsten et al. [25], in order to confirm effects at the group level.

These effects, being central in origin, presumably exclude peripheral metabolic factors or a pharmacokinetic explanation of the individual variability. Moreover, neither sex nor prior training history contributed to the behavioral variability in response to quinpirole (see Supplementary Results). We also measured D2-R and D1-R like receptor binding using autoradiography in these animals, but there were no relationships between quinpirole sensitivity and overall striatal D2-type or D1-type receptor binding signals or in the ratio between D1-type and D2-type receptor signals (Supplementary Results). The reasons for the individual variability thus remain obscure but may depend on subtle differences, such as hemispheric asymmetries in D2-R binding [36, 37]—some possibly suggestive evidence for this being reported in the Supplementary Results.

Our behavioral effects of quinpirole at low and mid-range doses were relatively selective and not the result of obvious sedative or motivational impairments. Baseline performance of visual discrimination was often intact in terms of errors committed, even at high doses of quinpirole. However, at the highest doses impairments were more disruptive, with prolonged response latencies, 4/6 animals stopping responding during reversal, and 1/6 failing to reach criterion on the baseline discrimination. The effects of quinpirole were determined 5 min after its administration, at a timepoint which produced a sub-maximal increase in locomotor activity in a preliminary dose-finding study (Supplementary Materials and Methods). Therefore, it would appear unlikely that the differential effects of the drug on baseline discrimination vs. reversal are simply due to time-course related actions of quinpirole.

Overall, this modulation of performance by quinpirole is entirely consistent with a tri-phasic profile, incorporating the so-called inverted U-shaped function. An inverted U-shaped curve has previously been invoked to explain chemical neuromodulatory effects on working memory in the prefrontal cortex [38, 39]. However, it is also consistent with data from human studies showing inverted U-shaped functions relevant to striatal functioning on the basis of both pharmacological and neuroimaging (PET) data [20, 40].

A serial reversal learning paradigm was employed because of its suitability for repeated drug treatments. After training, marmosets were able to reverse within a single session with an average of about 10 errors. These errors could not be attributed simply to response perseveration, but probably represent a mixture of learning and executive impairments. The choice of striatal site within the medial caudate nucleus for local infusions of quinpirole was based on previous findings for this species [6, 15]; this region is also in receipt of afferents from orbitofrontal cortical regions [41] implicated in reversal learning [42, 43]. However, other data indicate that the putamen may also be implicated in aspects of reversal learning [21, 44, 45] and the possible involvement of other striatal sites in the systemic effects of D2-type agonists like quinpirole cannot be excluded.

Given the limitations imposed by the extensive time necessary for training and ethical constraints on the number of infusions that could be administered, it was not feasible to perform a pharmacological analysis of these effects of quinpirole. This drug is also an agonist with quite high affinity at D3 and D4Rs in the primate striatum [46, 47]. Hence, it is possible that some of these effects of quinpirole depend on striatal D3 and D4Rs—members of the D2-like family of dopamine receptors [48]. Takaji et al.'s [22] use of viral knock-down of striatal D2Rs to impair reversal learning may help confirm a role specifically for the D2R.

There is growing evidence for a role for dopamine and D2-type receptors in reversal learning in humans, including patients with Parkinson’s disease [40, 49, 50]. Mehta et al. [51] found that the D2-type agonist bromocriptine impaired reversal learning in healthy volunteers at doses improving spatial working memory, suggesting different neural substrates for the optimal performance of these two tasks. Importantly, Cools et al. [40] found that baseline dopamine synthesis predicted the direction of effects of bromocriptine, which improved reward-based, relative to punishment-based, reversal learning in humans with low baseline dopamine synthesis capacity, while impairing reversal in subjects with high baseline striatal dopamine synthesis capacity. One problem with these human studies is that the lack of dose–response information makes it unclear whether such effects are produced by pre-synaptic autoreceptors or post-synaptic receptors and consequently whether the findings are due to increased or reduced striatal dopamine activity. The present findings suggest that D2-type agonists can exert differential effects on reversal learning via actions at both pre-synaptic and post-synaptic receptors.

This pharmacological evidence is counterpointed by genetic findings. den Ouden et al. [52] found that a DAT1 genotype-modulated human reversal learning, with computational modelling suggesting that an increasing number of 9R-alleles (thought to correspond with greater striatal dopamine transporter availability) resulted in a stronger reliance on previous experience and therefore a reluctance to update learned associations. Jocham et al. [19] showed that carriers of the A1 allele of the DRD2/ANKK1-TaqIa polymorphism, which is associated with reduced expression of D2Rs in the striatum, had deficits in a probabilistic reversal task.

Overall, the present demonstration of causal involvement of striatal D2-like receptors in reversal learning is evidently highly consistent across species and offers possible translational opportunities. For example, a previous study has shown that the D2-type agonist pramipexole enhances BOLD activity in the anterior caudate and improves reversal learning in stimulant drug abusers [13]. As reversal learning impairments are often present in schizophrenia [53, 54] and in Parkinson’s disease, possibly due to l-Dopa medication [49, 50, 55], it seems likely that striatal D2R-related mechanisms may play an important role in these cognitive deficits.

## Funding and Disclosure

All data supporting this publication are freely available at 10.17863/CAM.32242 as part of the University of Cambridge data repository. This work was supported by a Wellcome Trust Senior Investigator Award (104631/Z/14/Z to T.W.R.) and conducted within the University of Cambridge Behavioural and Clinical Neuroscience Institute, supported by a joint award from the Medical Research Council and the Wellcome Trust (MRC-G1000183). Funding to pay the open access publication charges for this article was provided by the Wellcome Trust.

## Electronic supplementary material

Supplemental Material
