# Supplementary material for: D2 receptors and cognitive flexibility in marmosets: tri-phasic dose–response effects of intra-striatal quinpirole on serial reversal performance
Source: Neuropsychopharmacology. 2018 Nov 15;44(3):564–71. doi: 10.1038/s41386-018-0272-9 (PMC6333796; doi:10.1038/s41386-018-0272-9)
Supplement: Supplementary file 1 — Supplemental Material [file 41386_2018_272_MOESM1_ESM.doc]

**SUPPLEMENTARY Materials and Methods**

**Subjects and housing**

Six common marmosets (*Callithrix jacchus;* 2 females, 4 males; mean ± SEM age at start of study = 3.0±0.28 years; at end = 5.8±0.32 years) – bred on site in a conventional barrier facility in the University of Cambridge Marmoset Breeding Colony – were housed in purpose-built housing (2.8m high x 1m^2^ floor space). Their cages contained a variety of environmental enrichment aids, including ropes, poles of varying diameters, and a nest box. Whenever possible, monkeys were housed in pairs. Colony rooms were maintained at 24°C and 55% relative humidity. Lights gradually illuminated the rooms from 07:00-07:30 and dimmed from 19:00-19:30 (12 hour light/dark cycle with dawn and dusk). All monkeys were fed MP(E) primate diet (Special Diet Services/SDS, Withams, UK) or egg and Complan sandwich (Nutricia Ltd., Trowbridge, Wiltshire, UK) and two pieces of carrot five days a week after the daily behavioral testing session, with simultaneous *ad libitum* access to water for two hours. At weekends, they had free access to water, and their diet was supplemented with fruit, rusk, malt loaf, treats, and egg and Complan sandwiches with vitamin supplements. Animals were regularly assessed by the Named Animal Care and Welfare Officer (NACWO) and the Named Veterinary Surgeon (NVS). All procedures were carried out in accordance with the UK Animals (Scientific Procedures) Act 1986 as amended in 2012, under project licences 80/2225 and 70/7618. In addition, the University of Cambridge Animal Welfare and Ethical Review Body (AWERB) provided ethical approval of the project licence and its amendments, as well as individual studies and procedures via delegation of authorization to the NACWO for individual study plans.

# Behavioral apparatus

Behavioral testing took place within an enclosed custom-built testing apparatus, with a house light illuminating the behavioral arena. As described previously (Clark*e et* al, 2008), the animal sat in a clear, plastic transport box, one side of which was removed to reveal a color, touch sensitive computer monitor (NEX121 TFT LCD Monitor, Nexio, Incheon, Korea). The marmoset reached through an array of vertical metal bars to touch stimuli presented on the monitor, and these responses were detected by the screen. Reward (cooled Nesquik banana powder in milk, Nestlé, York, UK) was delivered to a centrally placed spout by a peristaltic pump outside of the apparatus. Auditory stimuli were presented through standard PC speakers located at the back of the test chamber. Stimulus presentation upon the touchscreen, the speaker, and the reinforcer pumps was controlled by modules within the MonkeyCantab program (v8.1-11.3, R. N. Cardinal) developed from MonkeyCantab (Wee*d et* al, 1999; originally designed by T. W. Robbins and A. C. Roberts) using the Whisker control system (v4.0, Cardinal and Aitken, 2010) via an operant chamber interface (Biotronix, Cambridge, UK). Behavioral data were stored in a Microsoft Access (2003) database.

**Behavioral training**

*Pre-training phase*

All monkeys were trained to enter a clear plastic transport box for marshmallow reward and were habituated to the testing apparatus. Monkeys then received the following sequence of training: familiarization with banana-flavored milk reward, learning a tone-reward contingency, and responding on the touchscreen until they were reliably and accurately making at least 30 responses to a square stimulus pseudo-randomly presented to the left or right of the licker across a 30 min period (“Pre-training” in Figure 1B; for full experimental details, see Robert*s et* al, 1988).

*Reversal training phase*

As described previously (Clark*e et* al, 2004), serial discrimination reversal learning consisted of two-choice discriminations composed of abstract, multicolored visual patterns presented to the left and right of the center of the screen, with stimulus centers approximately equidistant from the licker and halfway up the screen. A response to the correct stimulus resulted in the incorrect stimulus disappearing from the screen and delivery of 5s of reinforcement. Failure to collect the reward was scored as a missed reinforcement. After a response to the incorrect stimulus, the correct stimulus disappeared from the screen, an audible negative reinforcer stimulus was played at 100dB for 0.25s, and the house light was extinguished for a 5s timeout period. The inter-trial interval was 3s. The relative position of visual stimuli varied across trials in a pseudorandom fashion, with each individual stimulus presented equally to the left or right of the reward licker across the session. If a monkey showed a significant side bias (10 consecutive responses to one side), a rolling correction procedure was implemented whereby the correct stimulus was presented on the non-preferred side until the monkey had made a total of three, not necessarily consecutive, correct responses. In order to compare the effect of multiple doses of a dopamine agonist infused into the caudate nucleus, animals were trained on reversal learning until they could reverse the contingencies within a single session (“Reversal Training” in Figure 1B). To facilitate this, the same set of stimuli was used throughout serial discrimination reversal testing.

Monkeys were initially trained to perform between-session reversals. Each monkey was presented with 30 trials per day, 5 days per week. The stimulus-reward contingency reversed after attaining a criterion of six correct responses in a row in the immediately preceding session. After each reversal, the previously correct stimulus became incorrect and the previously incorrect stimulus became correct. Once monkeys were reliably achieving six consecutive correct responses in daily sessions (i.e., contingencies reversed daily), a “baseline discrimination phase” was introduced at the start of each session. In this phase, marmosets were tested on the final discrimination from the preceding session before the contingencies were reversed. This allowed us to determine the effects of experimental manipulations on the reversal *per se*, independent of any potential impact on visual discrimination performance. After achieving a criterion of six correct out of seven responses, the contingencies reversed without any external signal (“reversal phase”). Successful within-session reversal was defined as six consecutive correct responses (Figure 1C). Failure to achieve this criterion resulted in completion of the reversal in the subsequent session. In this stage of testing, sessions ended following successful reversal, 2min inactivity, or 30min, whichever occurred first; there was no maximum trial limit for within-session reversals.

**Cranial cannulation surgery**

Subjects were pre-medicated with ketamine hydrochloride (0.1ml of a 100mg/ml solution, i.m.; Pfizer Animal Health, Sandwich, UK), given a 24h prophylactic analgesic (Carprieve; 5% w/v carprofen, s.c.; Norbrook Laboratories, Ltd., Corby, UK), and then intubated and maintained on isoflurane gas anaesthetic (flow rate: 2.0-2.5% isoflurane in 0.3 l/min O_2_; IsoFlo, Abbott Laboratories, Ltd., Maidenhead, UK), prior to being placed in a stereotaxic frame specially modified for the marmoset (David Kopf, Tujanga, CA, USA). Anesthesia was closely monitored clinically and by pulse oximetry and capnography.

Guide cannulae (C316G; Plastics One, Inc., Roanoke, VA, USA) were lowered at a 10° lateral angle (away from the midline), toward the target locations in medial caudate (n=6 animals; AP +12.5, LM ±2.2, V +12.0 from the interaural line). Guide cannulae were fixed in place using skull screws and cold cure dental acrylic (Simplex Rapid, Kemdent Works, Swindon, UK). The target coordinates were adjusted where necessary *in situ* according to cortical depth as described previously (Robert*s et* al, 2007). Postoperatively, all monkeys received an injection of dexamethasone (0.2ml of a 4mg/ml solution, i.m.; Organon Laboratories, Ltd., Cambridge, UK) to minimize tissue inflammation before being returned to their home cage for at least one week of “weekend diet” and *ad libitum* water to allow complete recovery before returning to testing. Monkeys were also given the analgesic Metacam (Meloxicam, 0.1 ml of a 1.5 mg/ml oral suspension; Boehringer Ingelheim, Ingelheim/Rhein, Germany) for three days post-operatively. Metal stylets covered with plastic dust caps (dummy cannulae, C316DC; Plastics One) were inserted and subsequently replaced on a weekly basis to maintain patency of guide cannulae.

**Habituation to infusion procedures**

Prior to each non-infusion session, marmosets experienced a mock infusion procedure, in which they were gently restrained by a person other than the experimenter in the infusion room. The experimenter tested the tightness of the cannula dust cap and operated the pump to mimic the infusion experience. Drug infusions commenced once animals acclimatized to these procedures, as indicated by stable behavioral testing after mock infusions.

**Intracranial infusion setup**

Drug solutions were delivered bilaterally by an infusion pump (KDS230, KD Scientific, Inc., Holliston, Massachusetts, USA) at a rate of 0.25µl/min via ~1m lengths of PTFE tubing (0.30mm inner diameter) connected to 10μl Hamilton microliter syringes (701RN; Hamilton, Bonaduz, Switzerland) with a blunt-tipped 22 gauge needle (Hamilton 7787-01) on one end and internal cannula (projecting 0.5-1.5mm from end of guide, depending on final depth of guide cannula; C316I, Plastics One) on the other by short lengths (<1cm) of solvent flexible tubing (0.38mm inner diameter, Elkay Laboratory Products, Ltd., Basingstoke, UK). Syringes, tubing, and internal cannulae were autoclaved or exposed to ethylene oxide sterilization.

**Locomotor activity pilot for selection of quinpirole doses used in the reversal study**

In order to determine a suitable dose range for intra-caudate quinpirole in the marmoset, a single marmoset (not included in the reversal study) was implanted with cannulae targeted at the medial caudate, and the effects of quinpirole on locomotion were assessed using a home cage activity assay (Figure S1). After bilateral infusion of quinpirole, the animal was immediately returned to the isolated upper right quadrant of the homecage and video-recorded for 60min. Time locomoting (in sec) was scored in 5-min bins using JWatcher (Version 1.01). Differences in locomotor activity were already evident in the first 5-min bin and guided the decision to test reversal animals 5min post-infusion. At this time point, the drug was clearly effective, but animals were not showing maximal locomotor effects.

**Autoradiography**

Following completion of infusions, marmosets were sedated with an injection of ketamine hydrochloride (0.1ml of a 100mg/ml solution, i.m.) before being terminally anesthetized with pentobarbital (1.0mL, 200mg/mL i.v., Vétoquinol UK Ltd., Buckingham, UK) and decapitated. Brains were rapidly excised and flash frozen in 2-Methylbutane (Sigma-Aldrich Chemie GmbH, Steinheim, Germany) cooled to -25°C using dry ice. All brains were stored at −80°C until use.

Autoradiographic binding for dopamine D2- and D1-type receptors was conducted using the same conditions as described previously (Jup*p et* al, 2013). A minimum of 10 serial 20μm coronal sections from around the cannula sites was collected using a cryostat and mounted onto super-frost plus slides (Menzel Glasser, Braunschweig, Germany). Sections were collected two per slide such that each slide contained sections from two animals. Slides were allowed to dry overnight before being stored at −80 °C.

Triplicate sections were warmed to room temperature prior to pre-incubation in 50 mm Tris–HCl buffer (pH 7.4; 30 min) followed by ligand incubation with either [3H]-raclopride (Perkin Elmer, MA, USA) for D2-type receptor binding or [3H]-SCH23390 (Perkin Elmer) for D1-type receptor binding. Non-specific binding for D2- and D1-type receptors was assessed in the presence, respectively, of haloperidol or flupenthixol (both from Sigma Aldrich, Dorset, UK) in one section for each animal. Excess ligand was removed through a series of washes in ice-cold buffer followed by ice-cold distilled water and left to dry over-night. Sections were then opposed to a tritium-sensitive phosphoimaging plate (Fujifilm, Tokyo, Japan) together with a [3H] microscale standard (Amersham Biosciences, Freiburg, Germany). Autoradiographs were digitised and a region-of-interest analysis conducted using Image J (Abràmof*f et* al, 2004) following calibration with microscale standards. Binding (in mol/mg tissue) for all ligands was assessed bilaterally in the medial caudate according to the region-of-interest in Figure S2. Specific receptor binding was calculated by subtracting non-specific binding from total binding for each region of interest. Results are presented in Table S1 as 1) average binding of duplicate sections for left and right hemispheres, 2) a ratio of D2-type to D1-type binding signal (which has previously been shown to relate to apomorphine-induced rotation biases in rats; Glic*k et* al, 1988), and 3) as the difference in binding signal between left and right hemispheres (i.e., left binding – right binding; hemispheric asymmetry in D2-type receptor binding in the putamen previously being implicated in differences between human subjects in sensitivity to reward vs. punishment and in incentive motivation; Tome*r et* al, 2008, 2014).

**Histological assessment of cannulation placements**

Cannulae placement was verified in the same 20-μm coronal sections that were assessed for autoradiographic dopamine receptor binding. Cell bodies were stained using Cresyl Fast Violet, and the sections were viewed under a Leitz DMRD microscope (Leica Microsystems, Wetzlar, Germany). For each animal, cannula locations were schematized onto drawings of standard marmoset brain coronal sections, and composite diagrams were then made to illustrate the extent of overlap between animals (Figure 2).

**Details of R analysis functions and packages**

Linear mixed-effects modelling was carried out using the “lmer” function from the “lme4” package (v1.1-13; Bate*s et* al, 2014) in R. Subsequent statistical tests (analysis of variance; ANOVA) were achieved using the “lmerTest” package (Kuznetsov*a et* al, 2017). Simultaneous general linear hypothesis testing (“glht” in “multcomp” package; Hothorn, 2017) was then applied with the Holm adjustment for multiple comparisons to further investigate any observed effects or interactions.

Fisher’s Exact Test (“fisher.test” in the “stats” package) was used to determine whether the incidence of failure to reach performance criterion differed between saline and “high” dose quinpirole conditions in the baseline discrimination and reversal phases of the task.

**SUPPLEMENTARY RESULTS**

**Within-session baseline and reversal performance remained stable across infusions**

The results of a three-way mixed model ANOVA (with factors of time point, day, and task phase) of raw error counts indicated stable behavioral performance across three time points (two sessions each at the beginning, middle, and end of the experiment). Performance at these time points also did not differ from the mean performance determined from the initial confidence limits analysis (Figure S3; see Figure 3A for confidence limits analysis). There was a main effect of task phase (F(1,75)=74.2, p=8.04x10^-13^), with subjects consistently making more errors in reversal than in the baseline discrimination phase (p=2.93x10^-5^ by general linear hypothesis test). There were no effects of or interactions between day or time point (F’s<1).

**Trial counts**

The dose-dependent pattern of trial difference scores was qualitatively similar to that seen for error difference scores (Figure S4). There was a significant dose by phase interaction (F(2,58)=6.37, p=0.0031) and a significant main effect of dose (F(2,58)=10.6, p=0.00012), but not of task phase (F(1,58)=0.0033), p=0.95). There was a significant effect of dose within the reversal phase (F(2,27.1=11.7), p=0.00022) but not the discrimination baseline phase (F<1). *Post hoc* analysis revealed this to be a significantly higher trial difference score at low doses (p=0.0015) as compared to saline and a significant difference between the effects of low- and mid-range quinpirole doses (p=8.2x10^-6^). However, the mid-range improvement did not reach significance (p=0.20).

**Response latencies**

A mixed model ANOVA was carried out on median response latency difference scores prior to correct and erroneous responses. There was a nearly significant prolongation of latency for correct responses (F(3,30.8)=2.61, p=0.070), which was driven by slower latencies at high compared to all other doses (p=0.08 in each case). Error latencies were significantly lengthened (F(3,34)=3.53, p=0.025), with high-dose quinpirole producing slower latencies than saline (p=0.04), low (p=0.012), and mid-range doses (p=0.04).

**Further investigation into the basis of inter-individual behavioral variability in sensitivity to intra-caudate quinpirole**

We investigated the potential contribution of several additional factors to the observed inter-individual behavioral variability after intra-caudate quinpirole infusions. Subjects were rank-ordered by lowest effective dose, most effective mid-range dose, and by dose that produced general disruption (Table S1) so as to look for potential patterns related to differences in sex, sessions to criterion in the reversal training phase, and autoradiographic measures of D2- and D1-type receptor binding. The only measure that appeared to relate to variability in response to intra-caudate quinpirole was a relationship between “lowest effective dose” and the difference in D2-type receptor binding between left and right hemispheres. Animals with a higher D2-type receptor binding signal in the left hemisphere were impaired at doses ranging from 0.003-0.03μg, whereas animals with low dose impairments starting at higher doses (0.3-1.0 μg) had a higher signal in the right hemisphere (denoted by red box and emboldened text in Table S1). This may be relevant to the previously published finding that hemispheric differences in D2-type receptor binding in another striatal region, the putamen, determine bias to human reward vs. punishment learning and incentive motivation (Tome*r et* al, 2008, 2014). However, as Subjects 1 and 3 did not receive quinpirole doses lower than 0.3μg, this result is only suggestive and requires further investigation in future studies.

**References**

Abràmoff MD, Magalhães, Paulo J, Ram, Sunanda J (2004). Image Processing with ImageJ. *Biophotonics International* **11**: 36–42.

Bates D, Mächler M, Bolker B, Walker S (2014). Fitting linear mixed-effects models using lme4. *arXiv:14065823 [statCO]* at <http://arxiv.org/abs/1406.5823>.

Cardinal RN, Aitken MRF (2010). Whisker: A client-server high-performance multimedia research control system. *Behavior Research Methods* **42**: 1059–71.

Clarke HF, Dalley JW, Crofts HS, Robbins TW, Roberts AC (2004). Cognitive inflexibility after prefrontal serotonin depletion. *Science* **304**: 878–880.

Clarke HF, Robbins TW, Roberts AC (2008). Lesions of the medial striatum in monkeys produce perseverative impairments during reversal learning similar to those produced by lesions of the orbitofrontal cortex. *J Neurosci* **28**: 10972–10982.

Glick SD, Lyon RA, Hinds PA, Sowek C, Titeler M (1988). Correlated asymmetries in striatal D1 and D2 binding: relationship to apomorphine-induced rotation. *Brain Research* **455**: 43–48.

Hothorn T (2017). *Simultaneous inference in general parametric models*. at <https://cran.r-project.org/web/packages/multcomp/multcomp.pdf>.

Jupp B, Caprioli D, Saigal N, Reverte I, Shrestha S, Cumming P, *et al* (2013). Dopaminergic and GABA-ergic markers of impulsivity in rats: evidence for anatomical localisation in ventral striatum and prefrontal cortex. *European Journal of Neuroscience* **37**: 1519–1528.

Kuznetsova A, Brockhoff PB, Christensen RHB (2017). *lmerTest: Tests in linear mixed effects models*. at <https://cran.r-project.org/web/packages/lmerTest/index.html>.

Paxinos G, Watson CRR, Petrides M, Rosa MG, Tokuno H (2012). *The marmoset brain in stereotaxic coordinates*. Academic Press: London, Waltham, Mass.

Roberts AC, Robbins TW, Everitt BJ (1988). The effects of intradimensional and extradimensional shifts on visual discrimination learning in humans and non-human primates. *The Quarterly Journal of Experimental Psychology Section B* **40**: 321–341.

Roberts AC, Tomic DL, Parkinson CH, Roeling TA, Cutter DJ, Robbins TW, *et al* (2007). Forebrain connectivity of the prefrontal cortex in the marmoset monkey (*Callithrix jacchus*): An anterograde and retrograde tract-tracing study. *J Comp Neurol* **502**: 86–112.

Tomer R, Goldstein RZ, Wang G-J, Wong C, Volkow ND (2008). Incentive motivation is associated with striatal dopamine asymmetry. *Biological Psychology* **77**: 98–101.

Tomer R, Slagter HA, Christian BT, Fox AS, King CR, Murali D, *et al* (2014). Love to win or hate to lose? Asymmetry of dopamine D2 receptor binding predicts sensitivity to reward versus punishment. *Journal of Cognitive Neuroscience* **26**: 1039–1048.

Weed MR, Taffe MA, Polis I, Roberts AC, Robbins TW, Koob GF, *et al* (1999). Performance norms for a rhesus monkey neuropsychological testing battery: acquisition and long-term performance. *Cognitive Brain Research* **8**: 185–201.

**Supplementary Tables**

|  | **Actual Dose (μg)** | **Subject** | **Sex** | **Reversal**  **Training Sessions** | **Mean Binding (mol/mg) (Both Hemispheres)** | | | **Left - Right Hemisphere** | |
| --- | --- | --- | --- | --- | --- | --- | --- | --- | --- |
|  |  |  |  |  | *D2R* | *D1R* | *D2R/D1R* | *D2R* | *D1R* |
| **Lowest effective**  **quinpirole dose** | 0.003 | 6 | m | 33 | 9.74 | 4.39 | 2.22 | **0.042** | 0.042 |
|  | 0.003 | 5 | m | 33 | 8.59 | 4.52 | 1.90 | **0.018** | -0.090 |
|  | 0.03 | 2 | f | 98 | 10.19 | 7.42 | 1.37 | **0.046** | 0.035 |
|  | 0.3 | 3 | f | 38 | 10.02 | 5.38 | 1.86 | **-0.001** | 0.144 |
|  | 0.3 | 1 | m | 81 | 6.60 | 4.22 | 1.56 | **-0.051** | 0.024 |
|  | 1.0 | 4 | m | 90 | 9.80 | 4.19 | 2.34 | **-0.046** | -0.022 |
| **Most effective mid-range quinpirole dose** | 1 | 1 | m | 81 | 6.60 | 4.22 | 1.56 | -0.051 | 0.024 |
|  | 1 | 3 | f | 38 | 10.02 | 5.38 | 1.86 | -0.001 | 0.144 |
|  | 1.0/3.0* | 6 | m | 33 | 9.74 | 4.39 | 2.22 | 0.042 | 0.042 |
|  | 3 | 2 | f | 98 | 10.19 | 7.42 | 1.37 | 0.046 | 0.035 |
|  | 3 | 5 | m | 33 | 8.59 | 4.52 | 1.90 | 0.018 | -0.090 |
|  | 6 | 4 | m | 90 | 9.80 | 4.19 | 2.34 | -0.046 | -0.022 |
| **Quinpirole dose producing**  **general disruption** | 3 | 3 | f | 38 | 10.02 | 5.38 | 1.86 | -0.001 | 0.144 |
|  | 3.0* | 6 | m | 33 | 9.74 | 4.39 | 2.22 | 0.042 | 0.042 |
|  | 10 | 1 | m | 81 | 6.60 | 4.22 | 1.56 | -0.051 | 0.024 |
|  | 10 | 2 | f | 98 | 10.19 | 7.42 | 1.37 | 0.046 | 0.035 |
|  | 10 | 4 | m | 90 | 9.80 | 4.19 | 2.34 | -0.046 | -0.022 |
|  | 10 | 5 | m | 33 | 8.59 | 4.52 | 1.90 | 0.018 | -0.090 |

**Table S1: Sex, reversal training sessions, and autoradiographic D2- and D1-type receptor binding rank-ordered by subjects’ lowest effective dose, most effective mid-range dose, and by the dose which produced general behavioral disruption.** *Subject 6 sustained irreparable implant damage, and thus the highest dose received was grouped with the dose producing general disruption and the two intermediate doses were taken for the most effective intermediate dose.

**Supplementary Figures**


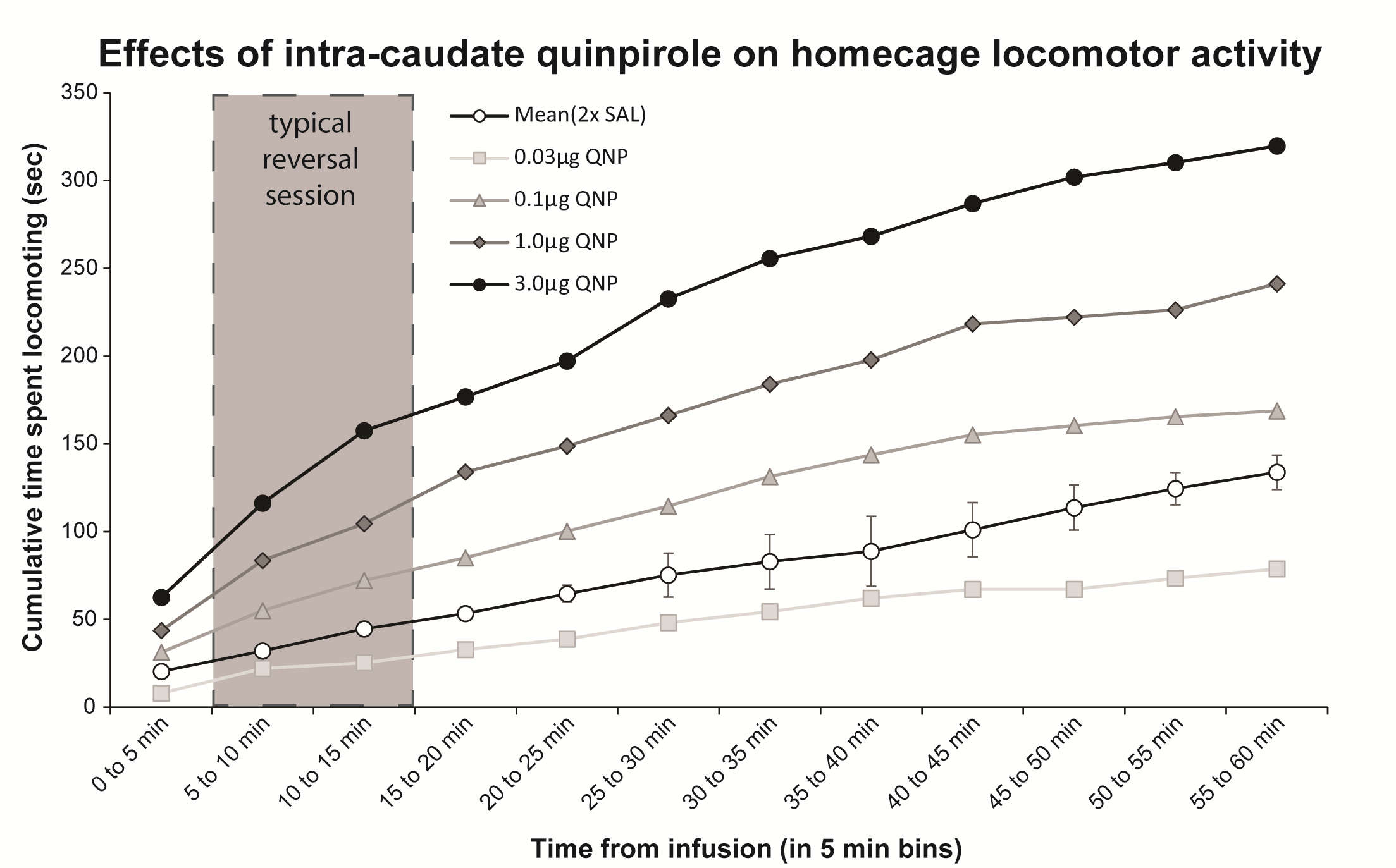


**Figure S1: Locomotor activity trial for establishing the initial intra-caudate quinpirole dose range.** Intra-caudate quinpirole was trialed in a single marmoset (not included in the reversal study) to establish the range and timing of doses likely to induce measurable behavioral changes. Time in motion (in sec) was scored in 5-min bins across a 60-min video-recorded session. The plot shows the cumulative number of seconds in motion and indicates a dose-dependent increase in locomotion at doses of 0.1μg and above and suppression at 0.03μg. The grey-shaded area bordered by a dashed line indicates the approximate length of a typical reversal session (median + IQR).


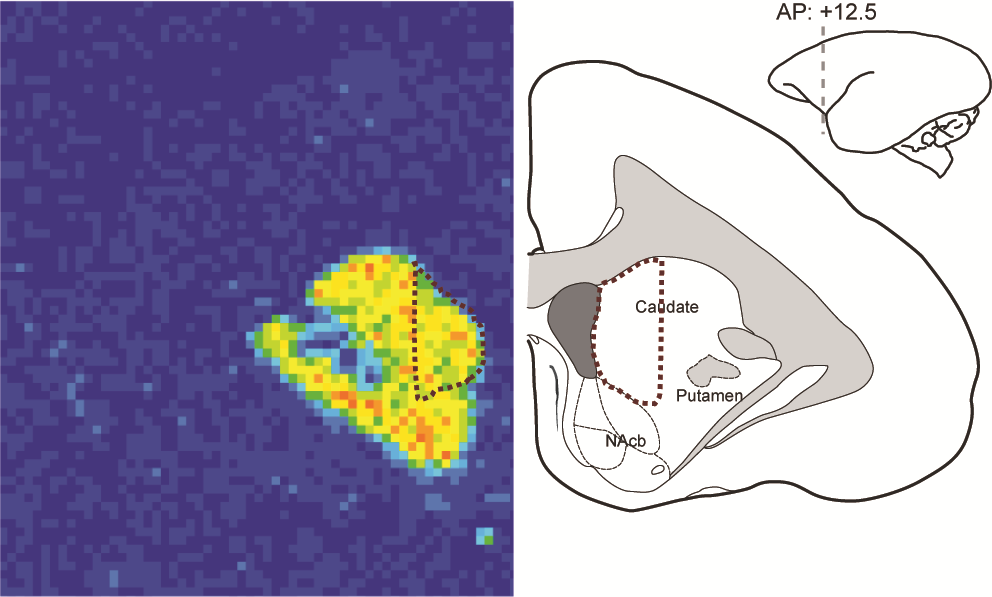


**Figure S2: Autoradiographic measurement of D2-type receptor binding: defining the region of interest.** Example autoradiograph of D2-type receptor binding from a single marmoset (left) and schematic image (right; adapted from (Paxino*s et* al, 2012) indicating the region of interest used for assessment of D2-type and D1-type receptor binding.


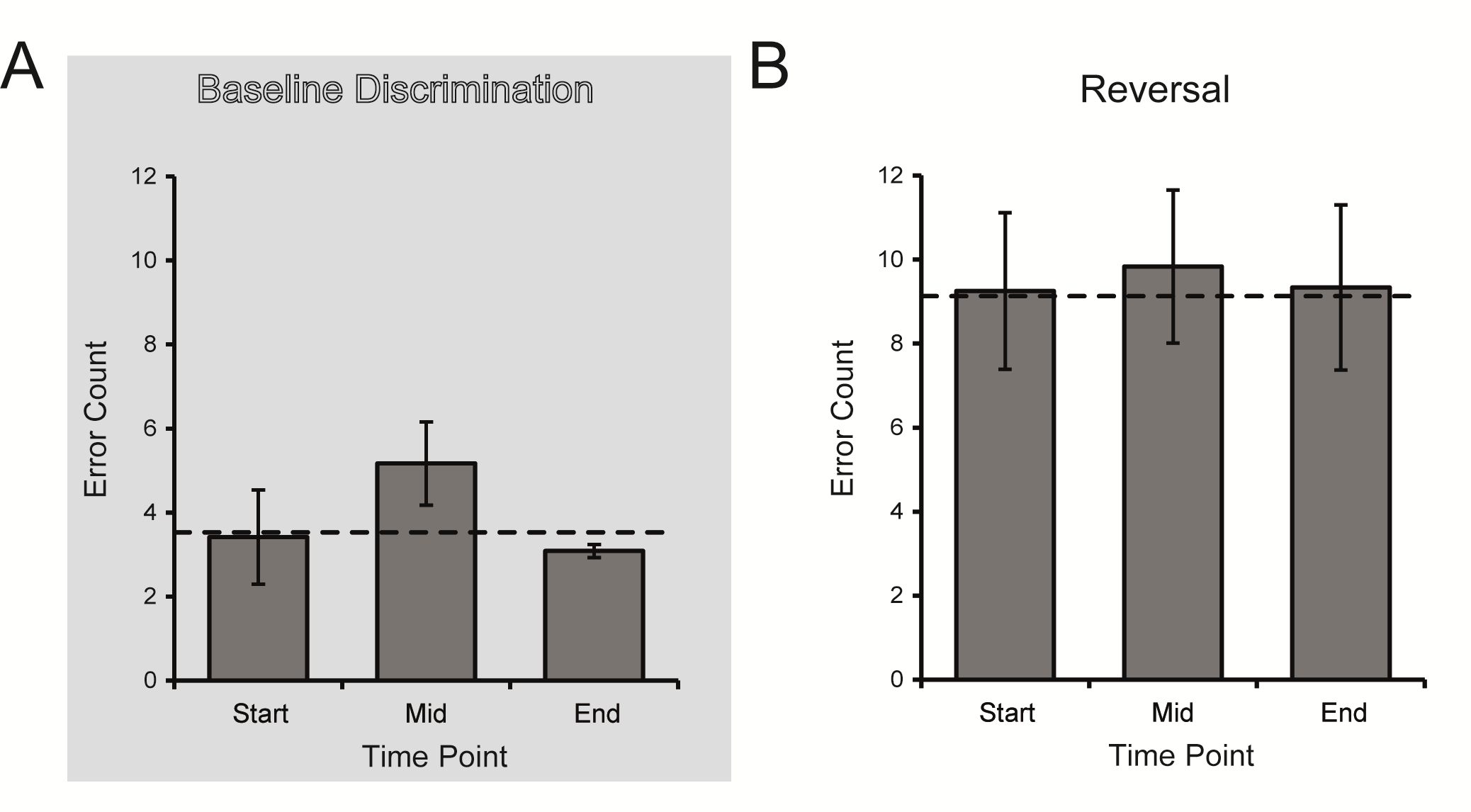


**Figure S3: Performance remained stable throughout the course of the experiment.** Performance (in terms of errors committed in baseline discrimination and reversal phases) was compared between three time points (two sessions each at the start, middle, and end of the study; grey bars) and the mean performance reported in the individual analysis (Figure 3A; dashed line in plots above).


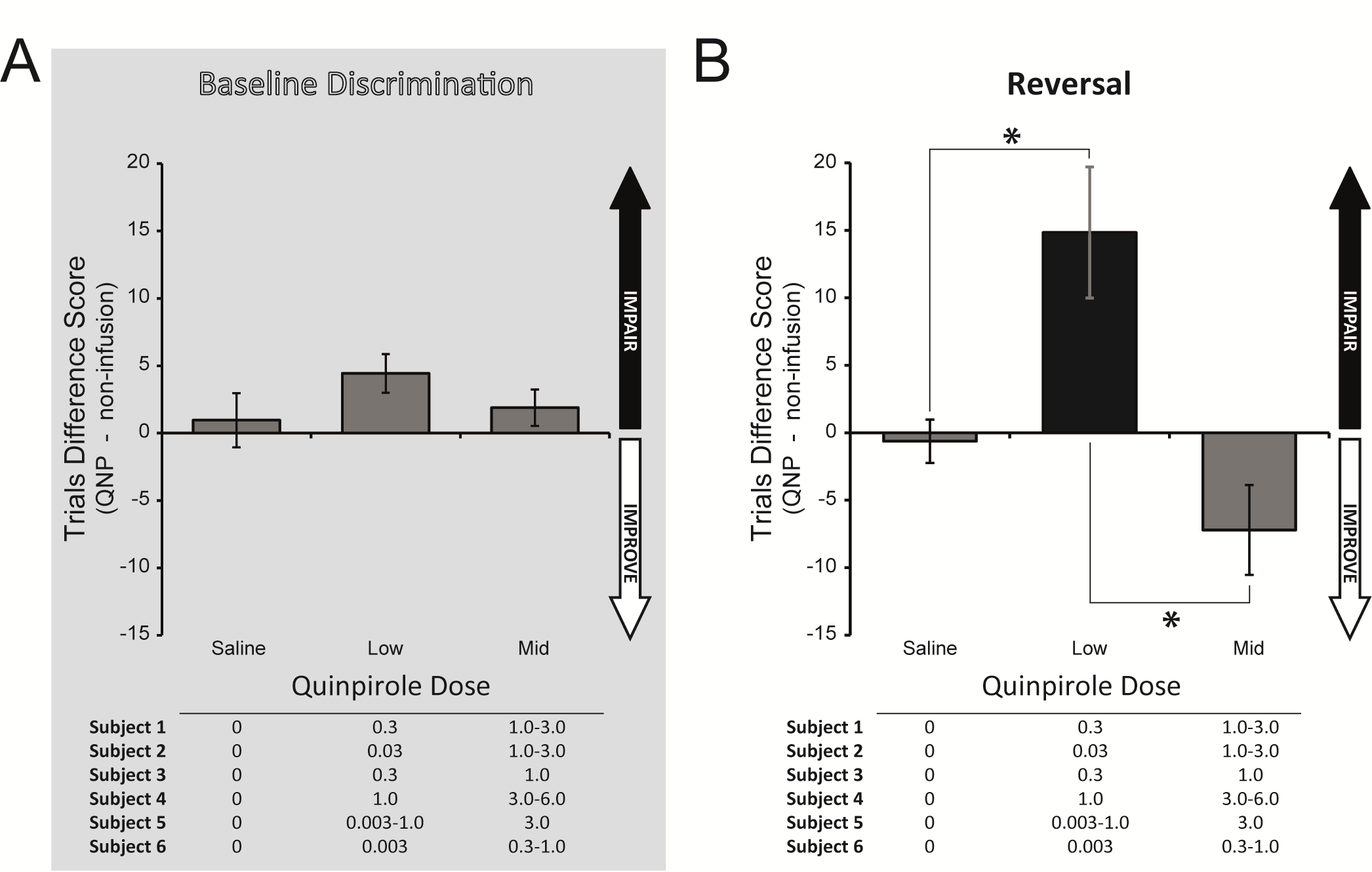


**Figure S4: Group analysis of trial data recapitulates the low-dose impairment effect from the error analysis.** Group summary and analysis of intra-caudate quinpirole effects on the trial difference score. Actual doses included in each dose category are shown in the tables below the bar plots. Low- and mid-dose effects were compared against the saline control infusion in a two-factor mixed model ANOVA. The dependent variable was a difference score between trials on the infusion day minus the mean trials of two preceding non-infusion sessions. There was a significant dose-dependent effect of intra-caudate quinpirole on reversal, but not baseline discrimination performance, with low-dose quinpirole inducing more errors. (* = p<0.05 by general linear hypothesis test following two-factor mixed model ANOVA.)
